# Design, Physical Characterizations, and Biocompatibility of Cationic Solid Lipid Nanoparticles in HCT-116 and 16-HBE Cells: A Preliminary Study

**DOI:** 10.3390/molecules28041711

**Published:** 2023-02-10

**Authors:** Ali Alamri, Ali Alqahtani, Taha Alqahtani, Adel Al Fatease, Saeed Ahmed Asiri, Reem M. Gahtani, Sulaiman Mohammed Alnasser, Jamal Moideen Muthu Mohamed, Farid Menaa

**Affiliations:** 1 Department of Pharmaceutics, College of Pharmacy, King Khalid University, Guraiger, Abha 62529, Saudi Arabia; 2 Department of Pharmacology, College of Pharmacy, King Khalid University, Guraiger, Abha 62529, Saudi Arabia; 3 Department of Clinical Laboratory Sciences, Faculty of Applied Medical Sciences, Najran University, Najran 61441, Saudi Arabia; 4 Department of Clinical Laboratory Sciences, College of Applied Medical Sciences, King Khalid University, Abha 61421, Saudi Arabia; 5 Department of Pharmacology and Toxicology, Unaizah College of Pharmacy, Qassim University, Buraydah 52571, Saudi Arabia; 6 Vaasudhara College of Pharmacy, Sante Circle, Chintamani Road, Hoskote 562114, Karnataka, India; 7 Departments of Medicine and Nanomedicine, California Innovations Corporation, San Diego, CA 92037, USA

**Keywords:** nanocarriers, cationic solid lipid nanoparticles (cSLN), cSLN-based delivery, cSLN-based therapy, cytocompatibility, hemolytic assay, MTS assay, surfactant

## Abstract

In this study, pEGFP-LUC was used as a model plasmid and three distinct cationic lipids (dioleyloxy-propyl-trimethylammonium chloride [DOTMA], dioleoyl trimethylammonium propane [DOTAP], and cetylpyridinium chloride [CPC]) were tested along with PEG 5000, as a nonionic surfactant, to prepare glyceryl monostearate (GMS)-based cationic solid lipid nanoparticles (cSLNs). Both the type and quantity of surfactant had an impact on the physicochemical characteristics of the cSLNs. Thermal analysis of the greater part of the endothermic peaks of the cSLNs revealed they were noticeably different from the individual pure compounds based on their zeta potential (ZP ranging from +17 to +56 mV) and particle size (PS ranging from 185 to 244 nm). The addition of cationic surfactants was required to produce nanoparticles (NPs) with a positive surface charge. This suggested that the surfactants and extensive entanglement of the lipid matrix GMS provided support for the behavioral diversity of the cSLNs and their capacity to interface with the plasmid DNA. Additionally, hemolytic assays were used to show that the cSLNs were biocompatible with the human colon cancer HCT-116 and human bronchial epithelial 16-HBE cell lines. The DOTMA 6-based cSLN was selected as the lead cSLN for further ex vivo and in vivo investigations. Taken together, these new findings might provide some guidance in selecting surfactants to prepare extremely efficient and non-toxic cSLN-based therapeutic delivery systems (e.g., gene therapy).

## 1. Introduction

Gene therapy has received more attention in recent years as a possible method to treat various inherited or acquired disorders. A promising solution for many uncommon genetic illnesses is gene therapy. To control the communication of transformed proteins liable for disease beginnings, nucleic acids (DNA and RNA) are introduced into target cells [1]. To exert efficient therapeutic effects, such genetic material must overcome many biological barriers to enter cells and interact with intracellular targets while avoiding destruction by nucleases that exist in plasma and the intracellular partition. In addition, nucleic acids have a low ability to cross cell membranes due to their high molecular weight and negative charge [2].

Consequently, a rationally designed and effective delivery method is crucial for successful gene therapy. Many vectors, categorized generically as viral and nonviral, have been developed for this purpose [3]. Owing to their site specificity and great transfection effectiveness, viral vectors were primarily considered as efficient carriers; however, their widespread clinical application is constrained by their immunogenicity and pathogenicity. Due to these drawbacks and concurrent breakthroughs in nanotechnology, numerous NP-DNA delivery systems (as non-viral vectors) have been prepared [4]. Due to their prolonged blood circulation patterns and several other technological advantages (e.g., high-scale production capacity and biocompatibility), unique and optimized delivery systems investigated lately have been suggested as non-viral carriers for gene therapy, namely lipid nanoparticles, particularly cSLNs [5,6].

By adding cationic surfactants to the lipid matrix, the relationships among delivery system elements and the genetic material (globally negatively charged) can be much improved [7]. So far, the cationic surfactant used in preparations has a significant impact on the physical and chemical properties of cSLNs, favoring complexation behavior toward intracellular penetration, genetic material, blood circulation-targeting characteristics, biocompatibility, and clearance rate [8]. The quantity ratio of lipid nanocarriers and material to be encapsulated for gene delivery requires careful consideration [9].

In the present study, using various types and concentrations of surfactants, we designed, constructed, and characterized several cationic cSLNs. Dioleyloxy-propyl-trimethylammonium chloride (DOTMA), cetylpyridinium chloride (CPC), and dioleoyl trimethylammonium propane (DOTAP) were chosen as the three quaternary ammonium salts to prepare new (to the best of our knowledge) and optimized cSLNs. They varied in terms of their cationic head groups and size of their hydrophobic carbon chains. In contrast to CPC and DOTAP, which each have just one hydrophobic carbon chain, DOTMA has two chains [10,11]. Subsequently, we investigated the physicochemical and biological characteristics of the prepared cSLNs affected by various types and concentrations of cationic surfactants. Owing to the different cationic surfactants utilized to form the pattern of the matrix, the biocompatibility and capacity of the prepared cSLNs to interface with plasmid DNA to produce stable complexes (cSLN-DNA) were specifically assessed.

## 2. Results and Discussion

### 2.1. Formulation and Characterization of cSLNs

**Figure 1:**
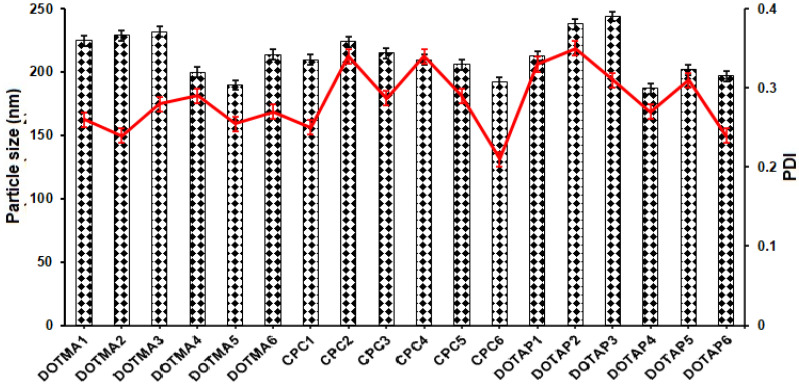
The average PS (nm) and PDI (red line) of the prepared cSLNs (*n* = 3; mean ± SD).

To determine if different concentrations and types of cationic surfactants had an impact on the characteristics of cSLNs, their capacity to form a complex plasmid DNA, and their biocompatibility, 18 formulations were successfully prepared. PEG 5000 was selected as the nonionic surfactant, while GMS was employed as the lipid matrix [12]. The tests were conducted using DOTMA, CPC, and DOTAP, three distinct cationic surfactants. The analytical makeup of the various cSLN formulations is displayed in Table 1. Before conducting the characterization, the formulations were thoroughly dialyzed against Milli-Q water to ensure purity. The resulting nanosystems were initially assessed for size and surface charge. All systems showed appropriate PDI values below 0.35, indicating NP uniformity [13], and an average PS in the nanometer scale, ranging from 185 to 244 nm (Figure 1). No discernible changes (*p* > 0.05) in PS or PDI were observed as the quantity of similar cationic surfactant increased, even though the quantity of cationic surfactant had a significant influence on the ZP values [14]. Kazeminezhad et al. (2012) described that a reduction in PS, a narrowing of the size distribution, and a geometrical change in particle from tetragonal, hexagonal, cubic, and triangular to quasi-spherical occurred with increasing surfactant concentration [15].

**Figure 2:**
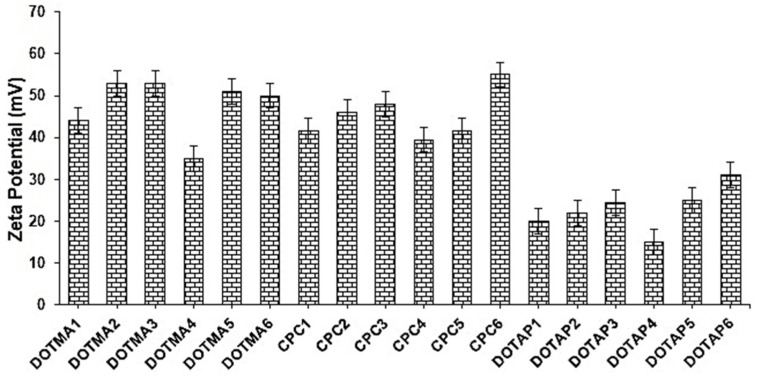
ZP values (mV) of the prepared cSLNs (*n* = 3; mean ± SD).

One of the properties of colloidal system stability is the surface charge, or ZP. It describes how strongly charged particles in a dispersion are attracted to one another [16]. A high ZP value corresponds to NP stability and prevents aggregation phenomena in the nanosuspensions; conversely, if the ZP is low, the dispersions will flocculate because attraction outweighs repulsion [17]. Figure 2 depicts the rise in surface charge values according to the amount of DOTMA, CPC, or DOTAP included into the NPs. Due to electrostatic attraction between the particles, the ZP values ranged from +17 to +56 mV, which were all positive, indicating a relatively high stability of colloidal dispersion [18]. Additionally, it was noted that the ZP values of the cSLNs made with DOTAP were much lower (*p* < 0.05) than those made with DOTMA and CPC, of which the nanosystems were not significantly different (*p* > 0.05).

### 2.2. DSC

**Figure 3:**
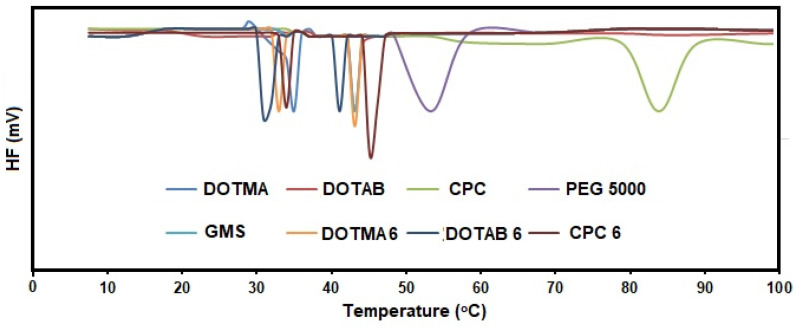
Thermograms of cSLNs (i.e., DOTMA 6, DOTAB 6, CPC 6) generated by DSC analysis compared with those of their pure constituents (i.e., DOTMA, DOTAB, CPC, PEG 5000, GMS).

To determine whether there was a direct relationship between cationic surfactant intercalation of the cSLN core and the polymorphic behavior of the cSLN matrix, DSC measurements on DOTMA-6, DOTAP-6, and CPC-6 were performed. Thermograms and the degree of crystallinity for the pure components—DOTMA, DOTAP, CPC, PEG 5000, and lipids (GMS)—were utilized in this work as a baseline to assess how they interacted with one another in the cSLN matrices [19]. Thermal measurements of bulk cSLNs revealed endothermic peaks that differed considerably from the standards, suggesting a complete mixture of the surfactants with the lipid matrix (Figure 3).

**Figure 4:**
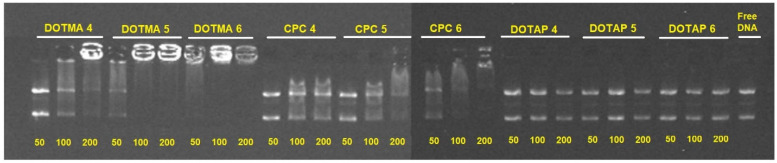
cSLN:pDNA complexes observed by agarose gel electrophoresis (0.8%) at 70 V for 45 min using three different weight cSLN:pDNA ratios (i.e., 50:1, 100:1, and 200:1 for trials 4, 5, and 6, respectively).

Some variations were observed because of the cationic surfactants utilized to formulate the cSLNs. In particular, for the surfactants CPC-6 (47 °C) and DOTAP-6 (38 °C), only one melting peak with a decrease in the residual crystallinity of the CPC and DOTAP phases was observed, which was attributed to the lipophilic crystalline core with respect to the pure surfactants (T = 61.5 °C, 62% and T = 58 °C, 87%, respectively), suggesting a complex formation (Table 2). For DOTMA-6, a more complicated thermogram was generated, and two distinct crystalline domains containing lipids were clearly visible at 41.5 and 54.4 °C (Figure 4). It was evident that there were five enthalpic contributions, each of which could be attributed to the fusion of the PEG 5000 (33.1 and 41.3 °C), DOTMA crystalline domains, lipid/DOTMA (44.5 °C), and lipid (52.8 °C). Notably, the mix still maintained the degree of crystallinity of the DOTMA (Table 2), indicating that contact between the neighboring hydrocarbon chains persisted even when the subsequent cSLN matrix was formed [20].

Overall, it was evident that DOTMA was packed differently in the cSLNs than the other surfactants with a single aliphatic chain, which resulted in variable interface behavior at the oil/aqueous interface. This circumstance should be favorable for an SLN formulation since it offers more stability with a lower rate of polymorphic transitions, but in this specific instance, DOTMA was not anticipated to behave as a solid lipid [21].

### 2.3. pDNA Binding Capability

By using three distinct cSLN/pDNA weight ratios (i.e., 50:1, 100:1, and 200:1), the impact of the various cationic surfactants (DOTMA, DOTAP, and CPC) on the ability of the cSLNs to bind pDNA was assessed. For the subsequent complexation with the pDNA, the nanosystems with the highest concentration of PEG 5000 (trials 4, 5, and 6) and best storage stability in terms of PDI were selected [22]. The cSLNs containing DOTMA produced the highest complexation efficiency, as seen in Figure 4. It is conceivable that because DOTMA has two lipophilic chains in comparison to CPC and DOTAP (C-18), a stronger positive surface charge would be contained inside the lipid network of the cSLNs, thus improving their ability to contact and electrostatically interact with the pDNA [23]. For cSLNs DOTMA-4, DOTMA-5, and DOTMA-6, 100% binding was attained at mass ratios of 50:1, 100:1, and 200:1, respectively. It was shown that the cationic surfactant content in the nanosystem had a direct impact on the ability of the cSLNs to form complexes, and that this effect was particularly pronounced as a function of the increased amount of cationic surfactant employed in the preparation [24].

The ZP values of the cSLNs containing DOTMA and CPC and their capacity to complex pDNA were not similar. The existence of other interactions, such as hydrophobic forces, between the pDNA and the more lipophilic surfactant DOTMA may have been responsible for this phenomenon [25]. Additionally, for the particles containing CPC, the quantity of cationic surfactant utilized in the preparation of the cSLNs had a direct correlation with how well the NPs interacted with the nucleic acid. The cSLNs containing DOTAP, on the other hand, failed to fix pDNA at some of the mass ratios examined. This may be explained by the fact that DOTAP confers a low surface charge (Figure 2), which was insufficiently positive to form electrostatic interactions between the particles and the pDNA [26].

### 2.4. Hemolysis Evaluation

Before being approved for administration to patients, a formulation meant for biomedical use must pass biocompatibility testing. For this reason, one of the procedures most employed to examine how NPs interact with blood components is the assessment of their hemolytic capabilities [27]. Hemolysis experiments were conducted by incubating cSLNs with human erythrocytes under controlled conditions and calculating the quantity of hemoglobin that was ultimately liberated by subsequent lysis of red blood cell surfaces. Erythrocytes were treated with Triton X-100 (1%) and DPBS (pH 7.4) to obtain results equivalent to 100% and 0% lysis, respectively.

**Figure 5:**
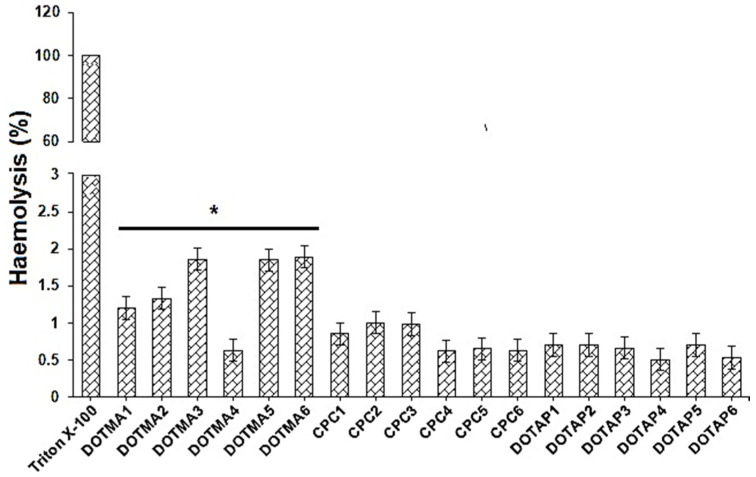
Hemolysis test after incubating cSLNs with red blood cells (RBC or erythrocytes) for one hour at 37 °C. RBC lysis was not evident in any of the samples. Data from three different experiments are shown as mean ± standard deviation. * *p* < 0.05 as compared to CPC and DOTAP.

Figure 5 displays that none of the cSLNs at the tested doses caused substantial red blood cell lysis, with less than 2.0% of the measured hemolysis percentage [28]. Indeed, when the hemolysis rate is <5%, a nanosystem can be considered as non-hemolytic [29]. Even though none of the examined cSLN samples were hemolytic, the greatest % hemolysis values were discovered for cSLNs containing DOTMA, and they were particularly related to the quantity of DOTMA in the formulations. When the erythrocytes were incubated with the other nanosystems (i.e., CPC and DOTAP), this behavior was not observed. Observations of all trials following incubation with the NPs revealed no erythrocyte aggregation. These findings showed that under the selected experimental circumstances, none of the prepared cSLNs caused lysis of red blood cell membranes [30].

### 2.5. In Vitro Metabolic/Cell Viability Assay

**Figure 6:**
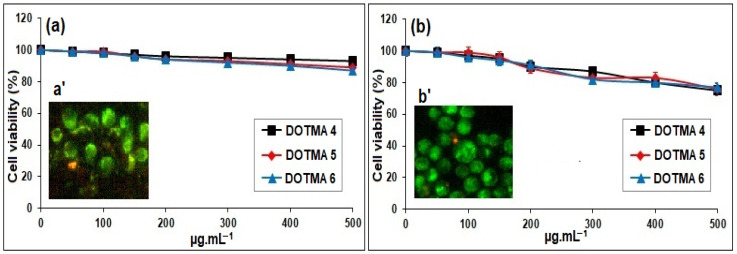
In vitro cell viability/metabolic activity profiles of DOTMA 4 (black), DOTMA 5 (red), and DOTMA 6 (blue)-based cSLNs on HCT−116 (**Panel a**) and 16−HBE (**Panel b**) cell lines. No significant differences were observed between these selected trials (*p* > 0.05). LIVE/DEAD stained HCT−116 (**Panel a’**) and 16−HBE (**Panel b’**) cells treated with DOTMA 6 by fluorescent microscopy (500 mg·mL^−1^).

Only the DOTMA-based cSLNs, which revealed 100% binding with pDNA, were used for the in vitro metabolic assay. After a 24-h period of incubation, the MTS assay was used to assess the cytotoxic potential of the DOTMA-based cSLNs, specifically DOTMA 4, DOTMA 5, and DOTMA 6, on HCT-116 and 16-HBE cells. Figure 6 displays the viability of HCT-116 (Figure 6a) and 16-HBE (Figure 6b) cells after being exposed to increasing concentrations of cSLNs. There was no discernible dissimilarity among normal and cancer cells in the pre-established range of concentration and incubation time and all the tested samples had confirmed mitochondrial activity, which can be directly related to cell viability [31]. Additionally, as the three curves appeared to be stacked, the dose-dependent cell viability trend that was observed in the hemolysis results was independent of the concentration of DOTMA present in the cSLNs. It should be noted that fluorescent micrographs of HCT-116 (Figure 6a’) and 16-HBE (Figure 6b’) cells stained with LIVE/DEAD dye after being exposed to greater concentrations of cSLNs (500 μg·mL^−1^) showed that the cells were virtually entirely alive (green fluorescence), in accordance with the MTS findings [32].

### 2.6. Storage Stability

One of the main issues for the quality of the finished product is the stability of cSLN dispersions. During storage, cSLNs with the PS (nm) of systems have a discernible propensity to combine and lose their physicochemical features [33]. As a result, the stability of the lead cSLN trial (DOTMA 6) was assessed in terms of PS, PDI, and surface charge while it was stored in dispersion at 4 °C for 30, 45, and 60 days in the dark (Table 3). Additionally, the formulations were stable for up to 60 days while preserving their physicochemical characteristics (data not shown). Notably, the samples with the lowest amounts of PEG 5000 (trials 1, 2, and 3) showed a minor rise in PDI values, demonstrating the importance of surfactant content in ensuring the shelf-stability of nano formulations [34]. This outcome suggested that the cSLNs had good physical stability when stored at 4 °C.

## 3. Materials and Methods

### 3.1. Materials

All the chemicals were analytical grade and utilized directly out of the container. Polyethelene glycol 5000 (PEG 5000), agarose, cetylpyridinium chloride (CPC), dioleyloxy-propyl-trimethylammonium chloride (DOTMA), and dioleoyl trimethylammonium propane (DOTAP) were all acquired from Sigma Aldrich (Bangalore, India). A free sample of glyceryl monostearate (GMS) was provided by Gattefossè (Mumbai, India). We bought the pEGFP-LUC plasmid from Addgene (Piscataway, NJ, USA). Gel Red™ Nucleic Acid Gel Stain obtained from BIOTIUM Inc. (Hayward, CA, USA) was used diluted 10,000× in water.

### 3.2. Preparation of cSLN

The ethanolic precipitation method was used to prepare the cSLNs, which were composed of solid lipid GMS and the surfactants PEG 5000 and DOTMA or DOTAP or CPC (Table 1). In brief, 150 mg of GMS was heated 5 to 10 °C beyond the melting point (m.p. 60 °C). The melted lipid phase was then mixed with an ethanolic PEG 5000 solution (215 to 315 mg; 2 mL) and the cationic surfactant. The subsequent organic solution was then dispersed into 100 mL of double-distilled water at 4 °C and homogenized using an Ultra Turrax T25 (IKA, Columbus, OH, USA) for 10 min at 13,500 rpm [35]. The nanosuspension was then refined thorough dialysis in a Spectra/Por1 dialysis tube with a 12,000 Da dialysis membrane (dialysis membrane-110, LA 395; Hi-media, Mumbai, India).

### 3.3. Particle Size (PS) Analysis

With the help of a Zetasizer Nano ZS (Malvern Instrument Ltd., Malvern, UK) [13], photon correlation spectroscopy (PCS) of the nanosuspension hydrodynamic diameter was performed and the PS distribution (PSD)-based polydispersity index (PDI) was studied. The experiment was carried out at a fixed angle of 180° in relation to the incident beam using the Zetasizer nano-associated non-invasive backscatter (NIBS) detection technology system, after the NPs were diluted to the proper concentration. The dispersing media used was double-distilled water (ddH_2_O) [36]. The results of the light scattering experiments are presented as the average values of triplicate trials (cSLN) from three distinct phases.

### 3.4. Zeta Potential (ZP)

Similar media and instrument settings used for PSD measurements were also used for ZP [37], and the analysis was carried out at 25 ± 0.5 °C with adequately diluted cSLNs [38]. The outcomes of these tests are shown as the mean values of triplicate trials from three distinct phases.

### 3.5. Differential Scanning Calorimetry (DSC) Analysis

During a change in temperature, DSC measures the quantity of heat excessively radiated or absorbed by the sample based on the temperature difference between the sample and reference material [39,40]. The DSC131 evo calorimeter (LabWrench/LabX, Midland, ON, Canada) was used to determine the surfactant’s ability to interact with the lipid matrix GMS (Mumbai, India). DSC measurements were performed using 2 mg of dried material placed in an aluminum crucible under the following conditions: a nitrogen atmosphere with a flow of 1 mL·min^−1^, a heating rate of 10 °C/5 min, and a heating range of 10 to 120 °C [41]. By deducting the baseline acquired for the reference standard and applying the parallel incorporation approach, the enthalpy for each transition shown in the thermograms was computed.

### 3.6. pDNA Binding Proficiency

The prepared cSLNs were assayed using an agarose gel electrophoresis gel retardation assay to determine their ability to bind pDNA. The pEGFP plasmid DNA was immediately combined with the diluted aqueous cSLN solutions. Different weight ratios (50:1, 100:1, and 200:1) using 5 µL of cSLN and 5 µL of DNA (5 ng·µL^−1^) were mixed and specifically examined at room temperature (RT) for 60 min. In order to observe the DNA for the DNA electrophoretic gel separations, 0.8% agarose in 1× TAE buffer (EDTA 1 mM and Tris acetate 40 mM) was used with 0.5 mg·mL^−1^ of Gel Red™ stain. The agarose gel was electrophoresed using a Consort Electrophoresis Power Supply 800 Series (Sigma Aldrich/Merck KGaA, Darmstadt, Germany) at 70 V for 45 min. The gels were observed and measured in a BioRad Gel Doc station using Quantity One software [42].

### 3.7. Hemolysis Assay/Hemocompatibility

Fresh K3 EDTA-treated blood was centrifuged at 2200 rpm for 10 min at 4 °C to isolate human erythrocytes (Research Ethics Review Board (ERB) approval no. 2022/18772/MedAll/TRY; dated 21 May 2022). The pellet was centrifuged 8× in Dulbecco’s phosphate-buffered saline (DPBS) with a pH of 7.4 before being resuspended in similar buffer with a final content of erythrocytes determined to be 4% [43]. This stock solution was continuously prepared from scratch and used within 24 h. A total of 500 mL of cSLN suspension was added to 500 mL of erythrocyte dispersion and shaken continuously during incubation at 37 °C for 1 h [44]. After subsequent centrifugation, the supernatants were subjected to a photometric study at 540 nm using a UV-Vis spectrophotometer (UV-1800, Shimadzu, Tokyo, Japan). Erythrocytes dispersed either in Triton (1% *w*/*v*) or DPBS (pH 7.4) were used for complete (100%) hemolysis (positive control) and zero hemolysis (blank/negative control), respectively. Each experiment was carried out thrice, in duplicate. The following formula was used to obtain the percentage of erythrocyte lysis:

Hemolysis (%) = (Abs preparation − Abs blank)/(Abs 100%lysis − Abs blank) × 100

### 3.8. In Vitro Metabolic/Cell Viability Assay

Using a readily accessible kit and following the manufacturer’s instructions, the MTS assay (Abcam, Cambridge, UK) was used to measure the cytotoxicity of the DOTMA-based series on human colorectal cancer (HCT-116) and human bronchial epithelial (16-HBE) cell lines. At a cell density of 2 × 10^4^ cells per well in a 96-well culture plate, the cells were cultured in DMEM with 10% FBS and 1% streptomycin/penicillin (10 mg·mL^−1^ streptomycin and 10,000 U·mL^−1^ penicillin) in a moistened atmosphere at 37 °C under 5% CO_2_. After 24 h, the medium was changed with 200 µL of new culture medium that included the lead cSLN at various doses (i.e., 100, 150, 200, 250, 300, and 500 µg·mL^−1^). The tests were performed on the plates after 24 h and 100 mL of freshly prepared medium with MTS solution (20 µL) was added. The absorbance was determined by microplate reader at 490 nm after an additional incubation of the cells for 2 h at 37 °C (iMark, Bio-Rad Laboratories Inc., Hercules, CA, USA). The negative control was cell medium. The % decline in the population of control cells was used to express the results [45]. The absorbance measured at 492 nm, stated as the average of six separate studies and SD, was inversely related to cell survival. Cells exposed to the higher concentration of 500 µg·mL^−1^ cSLN suspension were then rinsed with DPBS (pH 7.4), treated with the viable/non-viable fluorescent probe assay, and incubated at 37 °C for 10 min. An Axio Cam MRm fluorescence microscope (Axioscope 2plus, Carl Zeiss CMP GmbH, Goettingen, Königsallee, Germany) was then used to directly study cells.

### 3.9. Storage Stability

All the cSLN suspensions were kept in dispersion in the dark at 4 °C for two months (30, 45, and 60 days). They were described in terms of PS, PDI, and ZP at each prefixed time interval [46].

### 3.10. Statistical Analysis

The tests of each trial were accomplished in triplicate and the outcomes are displayed as mean and standard deviation (SD). The statistics were analyzed using one-way analysis of variance (ANOVA) with an appropriate significance level (*p* < 0.05). 

## 4. Conclusions

At this point, we showed that the mean PS of the cSLNs was below 250 nm and that the cationic surfactant concentration had a significant impact on the ZP values. The thermal studies of the bulk cSLNs revealed endothermic peaks that were considerably different from the targets, indicating full entanglement of the surfactants with the lipid matrix. On the other hand, DOTMA contains a double aliphatic chain compared to CPC and DOTAP surfactants, which resulted in variable interface behavior at the oil/aqueous interface in cSLNs. Hence, the more lipophilic surfactant DOTMA may be responsible for the pDNA-binding capability. The highest hemolysis percentage values were found for cSLNs containing DOTMA, and the fact that none of the tested cSLN samples were hemolytic also demonstrated 100% binding with pDNA. Therefore, the quantity of cationic surfactant introduced into the nano preparation had an impact on the efficiency of cSLN to form complexes, and the cSLNs with two lipophilic chains had the best complexation efficiency. Our findings indicated that these systems might be satisfactory non-viral vectors for gene therapy, while more biological research is necessary to validate our findings in ex vivo and in vivo settings (e.g., loading capacity of the molecule, evaluation of gene expression levels, effectiveness of the nanosystems). This work represents a step forward in the rational development and use of cSLNs for molecular encapsulation of nucleic acids (e.g., DNA, RNA, miRNA, siRNA). Considering SLN as a non-viral approach for safely treating a variety of disorders, we herein demonstrate that the rational incorporation of cationic lipids in SLNs will promote the binding of nucleic acids and, thus, such an cSLN could be used as non-viral vector for delivering one or a combination of genes. Nevertheless, to obtain a complete picture of this research, additional investigations are ongoing to estimate the transfection efficiency of the lead cSLN (i.e., DOTMA 4), the gene expression of some key markers and therapeutic genes, as well as the effectiveness of the lead cSLN (i.e., DOTMA 6) in an in vivo setting.

## Figures and Tables

**Table 1:** Methodical composition of the cSLN trials.

Preparation	Amount of Composition in mg				
	GMS	PEG 5000	DOTMA	CPC	DOTAP
DOTMA1	150.0	215.0	10	-	-
DOTMA2	150.0	215.0	20	-	-
DOTMA3	150.0	215.0	30	-	-
DOTMA4	150.0	315.0	10	-	-
DOTMA5	150.0	315.0	20	-	-
DOTMA6	150.0	315.0	30	-	-
CPC1	150.0	215.0	-	10	-
CPC2	150.0	215.0	-	20	-
CPC3	150.0	215.0	-	30	-
CPC4	150.0	315.0	-	10	-
CPC5	150.0	315.0	-	20	-
CPC6	150.0	315.0	-	30	-
DOTAP1	150.0	215.0	-	-	10
DOTAP2	150.0	215.0	-	-	20
DOTAP3	150.0	215.0	-	-	30
DOTAP4	150.0	315.0	-	-	10
DOTAP5	150.0	315.0	-	-	20
DOTAP6	150.0	315.0	-	-	30

GMS: glyceryl monostearate; PEG: polyethelene glycol; DOTMA: dioleyloxy-propyl-trimethylammonium chloride; CPC: cetylpyridinium chloride; DOTAP: dioleoyl trimethylammonium propane.

**Table 2:** Enthalpy values of the cSLNs for both pure and mixed compounds.

Preparations	CPC 6	DOTMA 6	DOTAP 6
	ΔH; J·g^−1^		
Pure PEG 5000	144.3	144.3	144.3
Blended	122.2	110.4	110.8
Pure GMS	147.6	147.6	147.6
Blended	177.8	152.3	151.6
Pure DOTMA	-	44.5	-
Blended	-	41.8	-
Pure DOTAP	-	-	153.6
Blended	-	-	28.7
Pure CPC	214.4	-	-
Blended	71.8	-	-

**Table 3:** Various stability parameters of the lead cSLN (DOTMA 6) at 4 °C for up to 60 days.

Evaluations	0 Month	30 Days	45 Days	60 Days
PS (nm)	183.44 ± 3.22	189.97 ± 4.01	198.63 ± 4.49	209.65 ± 5.21
PDI	0.221 ± 0.11	0.228 ± 0.15	0.233 ± 0.19	0.268 ± 0.23
ZP (mV)	34.7 ± 1.78	32.1 ± 1.62	28.7 ± 2.51	22.4 ± 3.77

Each value expressed as mean (*n* = 3; mean ± SD). No significant differences were observed in a given tested parameter for the lead cSLN (intragroup comparisons). The same letter indicates statistical insignificance (*p* > 0.05), as evaluated by ANOVA test.

## Data Availability

Data sharing not applicable.
